# A Novel *TRPC6* Mutation That Causes Childhood FSGS

**DOI:** 10.1371/journal.pone.0007771

**Published:** 2009-11-10

**Authors:** Saskia F. Heeringa, Clemens C. Möller, Jianyang Du, Lixia Yue, Bernward Hinkes, Gil Chernin, Christopher N. Vlangos, Peter F. Hoyer, Jochen Reiser, Friedhelm Hildebrandt

**Affiliations:** 1 Departments of Pediatrics and of Human Genetics, University of Michigan, Ann Arbor, Michigan, United States of America; 2 Department of Medicine, Division of Nephrology and Hypertension, Leonard Miller School of Medicine, University of Miami, Miami, Florida, United States of America; 3 Department of Cell Biology, University of Connecticut Health Center, Farmington, Connecticut, United States of America; 4 Klinik für Pädiatrische Nephrologie, Universitäts-Kinderklinik Essen, Essen, Germany; 5 Howard Hughes Medical Institute, University of Michigan, Ann Arbor, Michigan, United States of America; University of Cincinnati, United States of America

## Abstract

**Background:**

*TRPC6*, encoding a member of the transient receptor potential (TRP) superfamily of ion channels, is a calcium-permeable cation channel, which mediates capacitive calcium entry into the cell. Until today, seven different mutations in *TRPC6* have been identified as a cause of autosomal-dominant focal segmental glomerulosclerosis (FSGS) in adults.

**Methodology/Principal Findings:**

Here we report a novel *TRPC6* mutation that leads to early onset FSGS. We identified one family in whom disease segregated with a novel *TRPC6* mutation (M132T), that also affected pediatric individuals as early as nine years of age. Twenty-one pedigrees compatible with an autosomal-dominant mode of inheritance and biopsy-proven FSGS were selected from a worldwide cohort of 550 families with steroid resistant nephrotic syndrome (SRNS). Whole cell current recordings of the mutant TRPC6 channel, compared to the wild-type channel, showed a 3 to 5-fold increase in the average out- and inward TRPC6 current amplitude. The mean inward calcium current of M132T was 10-fold larger than that of wild-type TRPC6. Interestingly, M132T mutants also lacked time-dependent inactivation. Generation of a novel double mutant M132T/N143S did not further augment TRPC6 channel activity.

**Conclusions:**

In summary, our data shows that TRPC6 mediated FSGS can also be found in children. The large increase in channel currents and impaired channel inactivation caused by the M132T mutant leads to an aggressive phenotype that underlines the importance of calcium dose channeled through TRPC6.

## Introduction

Focal segmental glomerulosclerosis (FSGS) is the most common cause of steroid-resistant nephrotic syndrome in both children and adults [1]. The prognosis of untreated patients with FSGS is poor as 50–70% of patients will develop end-stage renal failure (ESRF) [2].

Mutations in podocin (*NPHS2*) and *PLCE1* cause autosomal-recessive FSGS, whereas autosomal-dominant FSGS is caused by mutations in *WT1*, *CD2AP*, *ACTN4* and *TRPC6* [3], [4], [5], [6], [7], [8], [9], [10]. Autosomal-recessive forms exhibit childhood-onset, whereas autosomal-dominant forms cause adult-onset nephrotic syndrome (NS). Monogenic disease genes of NS code for proteins that are essential for nephron development and an intact podocyte slit diaphragm [11].

Heterozygous mutations of *TRPC6* were recently identified to cause late-onset autosomal-dominant FSGS [9], [10], [12]. The *TRPC6* gene is located on the long arm of chromosome 11 (11q22.1) and codes for the short transient receptor potential canonical 6 (TRPC6). TRPC6 belongs to the family of transient receptor potential (TRP) channel proteins, a diverse group of voltage-independent cation-permeable channels that are expressed in many tissues including lung, kidney, brain, muscle containing tissues and blood cells where they are involved in many different signaling cascades of cell growth and mechanosensation [9], [13]. The TRPC subfamilies (TRPC1-TRPC7) are important for the increase of the intracellular Ca^2+^ concentration in response to activation of G-coupled protein receptors and receptor tyrosine kinases [14]. TRPC6 is a receptor-operated channel and is directly activated by diacylglycerol (DAG) responding to phospholipase C (PLC)-mediated signals [15]. In the kidney, TRPC6 is expressed in both renal tubules and glomeruli, with predominance in podocytes. TRPC6 is an essential component of the podocyte slit diaphragm, where it is integrated into a signaling complex that interacts with nephrin and podocin [10]. A recent study showed that induction of TRPC6 expression in cultured podocytes causes loss of actin stress fibers and proteinuria in mice, thereby suggesting that TRPC6, in podocytes, is functionally connected to the actin cytoskeleton [16]. The exact pathophysiology through which dysregulated Ca^2+^ influx in patients with *TRPC6* mutations leads to FSGS remains unclear. So far, seven different mutations in *TRPC6* were identified in seven different families from different ethnic backgrounds [9], [10], [12]. All patients manifested with disease between ages 18 and 57. Four of these seven mutations (R895C, E897K, P112Q and Q889K) allow significantly higher Ca^2+^ currents compared to wild-type TRPC6 Ca^2+^ current. Moreover, induction of wildtype TRPC6 protein expression is associated with higher cellular calcium during acquired proteinuria syndromes. These findings suggest high abundance of wild-type TRPC6 channels in podocytes contribute to a similar pathophysiology as the presence of mutated, overly active channels. The fact that both hyperactive mutated as well as high expression of wt TRPC6 can cause proteinuria syndromes with different onset time, suggests that the level of calcium available in the foot process is a determining factor for disease activity. Comparison of average inward and outward channel currents and inactivation time of all published mutations shows that all mutations lead to increased TRPC6 channel activity by one of the above mechanisms, M132T calcium peak influx is the largest calcium peak influx described so far (**Table 1**). The results from this paper supports the hypothesis of calcium dose-response dependency showing that a novel human mutation with largest calcium peak influx and strong delay of channel activity can lead to early FSGS.

**Table 1:** Genotype-phenotype correlation of all published mutations.

Mutation	Averaged inward current amplitude of the mutant channel	Average increase in inward current of mutant channel *vs* WT channel	Increase in Ca2+ currents or influx of mutant channel *vs* wt channel	Delayed channel inactivation	Activation by	Age at onset of disease (in years)	Reference
M132T	−701 pA/pF (−120 mV)	6.7	10	Yes**	CCh	9–30	current study
P112Q	−9 nA (100 µM UTP)	3	1.7	nd	UTP	18–56	9
P112Q	nd	nd	1.7	nd	Ang-2	18–56	9
R895C	−230 (−100 mV)	7.7	nd	No	CCh	18–46	10
E897K	−150 (−100 mV)	5	nd	No	CCh	24–35	10
N143S	−300 pA/pF (−120 mV)	2.1	-	No**	-	27–39	current study
S270T	−43.1 pA/pF	−0.6*	-	Yes**	CCh	17–52	10, current study
K874X	−112.3 pA/pF	0.08	-	Yes**	CCh	27–57	10, current study
Q889K	nd	nd	7,5	nd	OAG	35–41	12

CCh = Carbachol; UTP = uridine 5′-triphosphate; Ang-2 = Angiotensin II; nd = not done.

* S270 current was smaller than wt TRPC6. The inward currents ratio: N143s/WT = 2.1; K874X/WT = 0.08.

** M132T did not inactivate within 100 s. On average, WT and N143S inactivated by 60% within 10–20 s; whereas S270T and K874X inactivated by 10–15% within 79–90 s.

We performed mutation analysis of *TRPC6* in 21 familial cases with FSGS that were compatible with autosomal-dominant inheritance and identified a novel *TRPC6* mutation as the cause of FSGS. One patient manifested disease already at the age of 9. To study the effect of M132T on calcium channel function, we expressed wild-type, M132T and a newly generated double mutant M132T/N143S in HM1 cells (human embryonic kidney cells stably transfected with the M1 muscarinic receptor). The M132T mutant increased TRPC6 in- and outward Ca^2+^ currents significantly, compared to wild-type TRPC6 currents. In contrast to the wild-type TRPC6 current, the mutant M132T did not show inactivation after reaching a peak level upon activation, and only a minor decline of inward current was observed, representing a new variant of TRPC6 dysfunction.

## Results

### Patient Characteristics

We ascertained 52 individuals with FSGS from 21 families in whom disease segregation was compatible with an autosomal dominant pattern. At least one affected individual in each family had biopsy proven FSGS. We identified 14 families with two affected members, five families with three affected members, one family with four affected members and one family with five affected members (**Table S1**). No patients had mutations in *NPHS2* and *WT-*1. Age at onset of disease was documented in 38 patients. The median age at onset of disease was 17.5 years (range 2–70 years). Patient recruitment for this study was worldwide with a predominance of (Central) European individuals. Clinical characteristics are listed in **Table S1**.

### *TRPC6* Mutation Analysis

**Figure 1:**
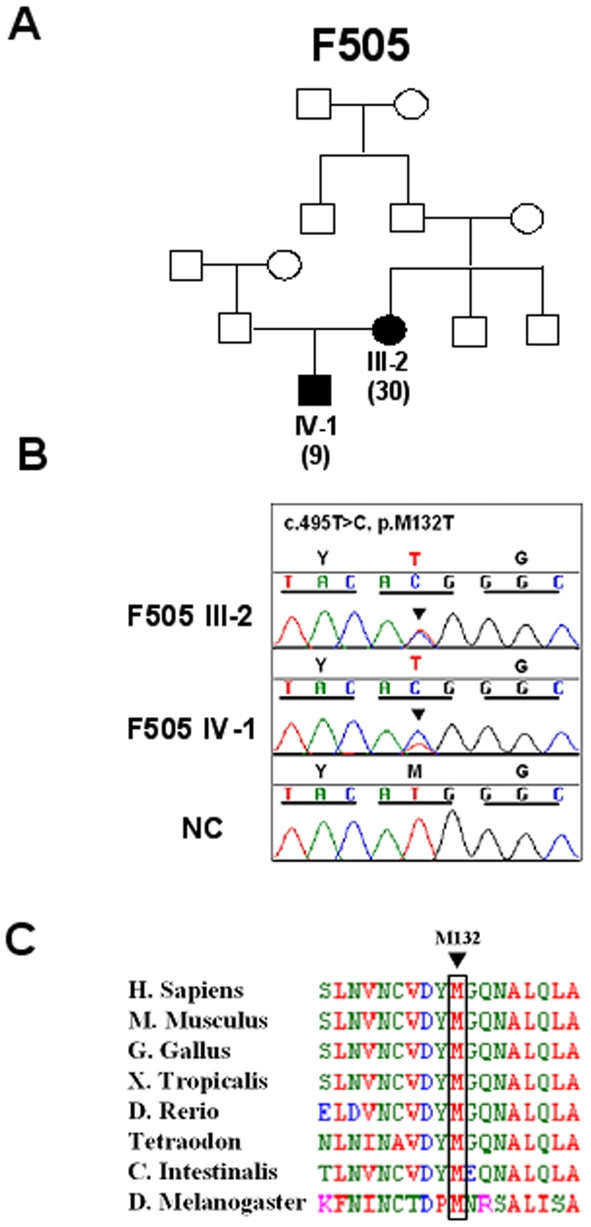
Segregation and Evolutionary conservation of M132T. (**A**) Pedigree of FSGS Family F505 with affecteds III-2 and IV-1 is compatible with autosomal/dominant inheritance. Filled symbols denote individuals affected with FSGS. Age at onset (years) is shown in parentheses. (**B**) *TRPC6* mutation in F505. Nucleotide and amino acid change for M132T are given above sequence traces. The normal control (NC) sequence is shown below the mutated sequence. Reading frame is indicated by underlining all codon triplets in the chromatogram. (**C**) Evolutionary conservation of *TRPC6* The Met^132^ is highly conserved through evolution down to *Drosophila Melanogaster*.

Mutation analysis of *TRPC6* by direct exon sequencing of all 13 exons was performed in at least one affected individual with biopsy proven FSGS from all 21 selected families. In family F505, a two-affected family from Turkish descent, we detected a novel *TRPC6* mutation which segregated with disease (**Figure 1A**). Family member III-2 manifested disease at 30 years of age and has biopsy proven FSGS. Her son, IV-1, manifested disease at the age of 9 and also has biopsy proven FSGS. Both affecteds had steroid-resistant nephrotic syndrome (SRNS) and developed end-stage renal failure 0.5 (III-2) and 9.5 (IV-1) years after onset, respectively.

The mutation changes a T to a C in exon 5 (c.495T>C), which substitutes a Methionine to a Tryptophan (M132T) (**Figure 1B**). This amino acid is highly conserved through evolution even in *Drosophila Melanogaster* (**Figure 1C**) and is located in the second intracellular ankyrin repeat of the TRPC6 protein. The mutation was absent from >140 healthy control individuals.

The following innocuous sequence variants that represent known single nucleotide polymorphisms (SNPs) were found: P15S; SNP rs3802829 was found in seven patients A404V; SNP rs36111323 was found in seven additional patients. All of the other sequence variants that were found in this cohort (p.N561N, p.Y705Y, p.F843F and p.Q904Q) did not result in an amino acid change.

### Electrophysiological Analysis of Mutant and Wild-Type TRPC6

**Figure 2:**
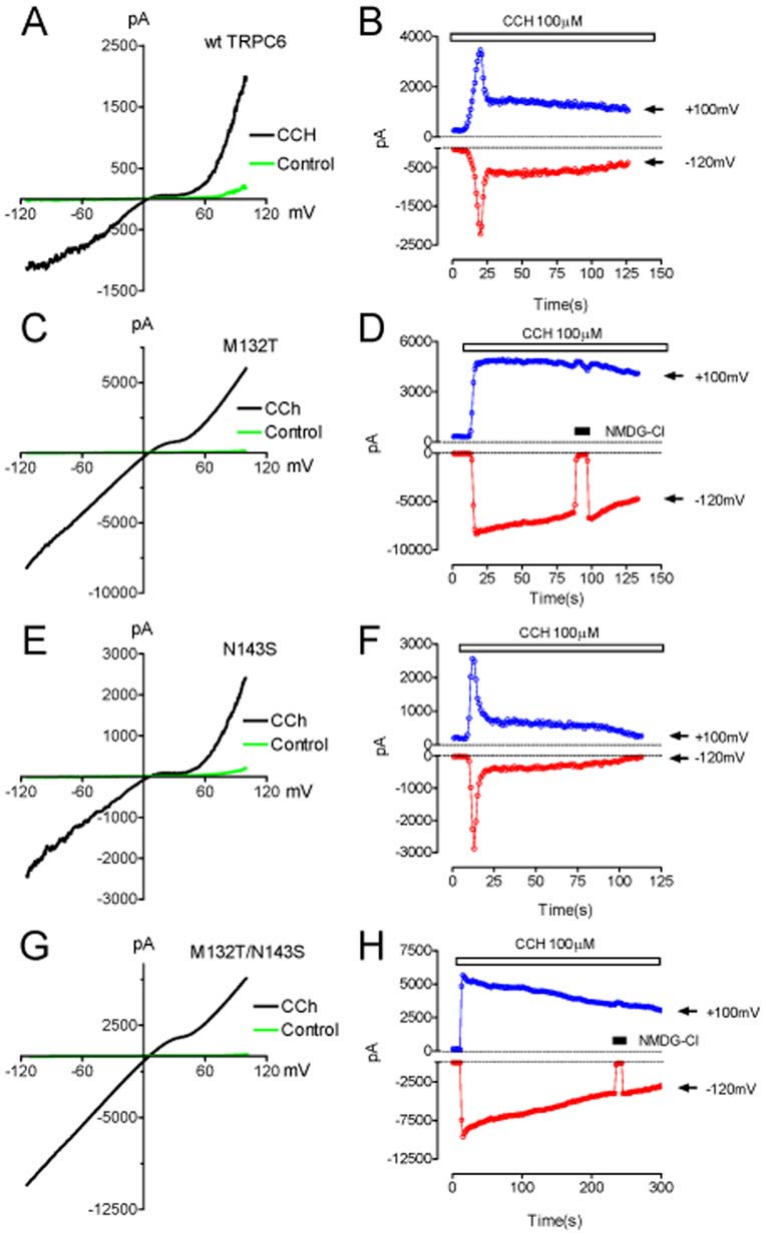
Gain of function of TRPC6 mutants M132T and M132T/N143S. **A**, **C**, **E**, and **G**: Representative currents elicited by voltage-ramp protocol ranging from −120 to +100 mV in the presence of 100 µM Carbachol (CCh) in wt TRPC6 (**A**), M132T (**C**), N143S (**E**), and M132T/143S (**G**). No activation was observed before 100 µM CCh stimulation. **B**, **D**, **F**, and **H**: Time-dependent changes of inward current measured at −120 mV and outward current measured at +100 mV of wt TRPC6 (**B**), M132T (**D**), N143S (**F**), and M132T/143S (**H**). TRPC6 and the mutants were only activated after application of 100 µM CCH. In both panel **D** and **H**, N-methyl D-glucamine (NMDG) was applied to confirm that there was no leak current. For panel **B** and **F**, there was no significant difference of the sustained current amplitude between wt TRPC6 and N143S.

**Figure 3:**
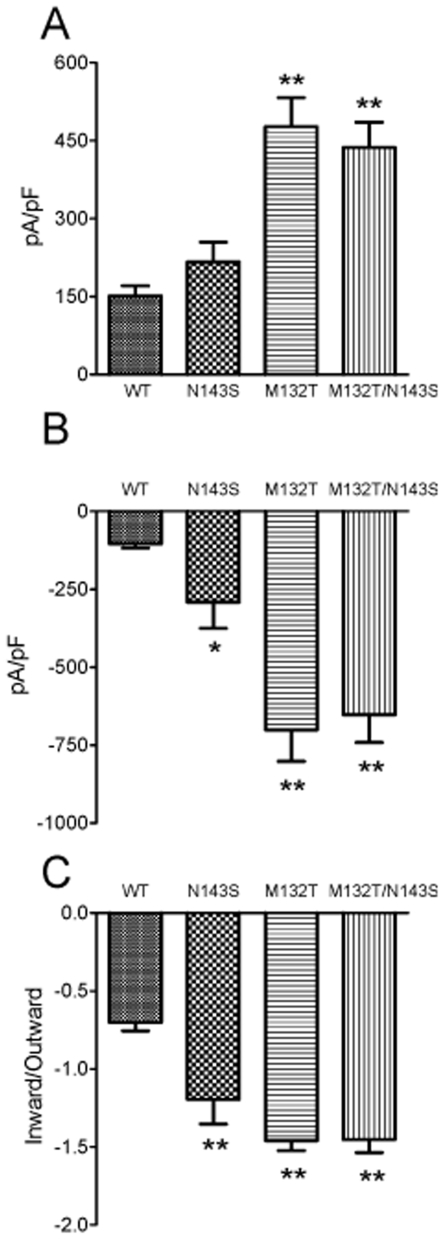
Averaged current amplitude of wt TRPC6 and the mutants. **A**: Outward current amplitude measured at +100 mV (mean±SEM, n = 10). The outward current amplitude of M132T and M132T/N143S was significantly bigger than that of wt TRPC6. **B**: Inward current amplitude measured at −120 mV (mean±SEM, n = 10). The inward current amplitude of N143S, M132T, and M132T/N143S was statistically larger than that of wt TRPC6. **C**: The ratio of inward/outward current amplitude. Note the current amplitude of wt TRPC6 and N143S was measured at the peak inward and outward current amplitude (as shown in Figure 2). (*: p<0.05, **: p<0.01, in comparison with wt TRPC6).

In order to access the functional significance of the M132T mutation, TRPC6 current was recorded after 36–48 hrs transfection by a voltage ramp protocol ranging from −120 to +100 mV. **Figure 2A** shows wt TRPC6 current elicited by 100 µM Carbachol (CCh). The time-dependent changes of the current amplitude (**Figure 2B**) indicated that TRPC6 was rapidly activated and reached a peak level after the cell was stimulated by 100 µM CCh. TRPC6 current then declined to about 50% of the peak current level and maintained at a sustained current level. No current was activated before application of CCH (**Figure 2A**). Interestingly, the mutant M132T exhibited significantly increased current amplitude (**Figure 2C**). The average outward and inward current amplitudes of M132T were almost 3- and 5-fold bigger than that of wt TRPC6 (**Figure 3**). The second remarkable difference between M132T and the wt TRPC6 was that M132T did not show inactivation and only minor time-dependent decline of inward current was observed (**Figure 2D**). The position of M132T is close to the previously reported mutation N143S and occurs within the same ankyrin domain [10]. To test if M132T has reached a limit for gain-in function, we created a TRPC6 double mutant M132T/N143S in order to study positive additive effects on channel kinetics. We first tested the properties of the known FSGS mutant TRPC6-N143S alone. As shown in **Figure 2E–F**, N143S displayed similar rapid activation and inactivation as observed in wt TRPC6. However, the peak inward current amplitude of N143S was bigger than that of wt TRPC6. The peak outward current amplitude as well as the sustained currents (after inactivation) of N143S were not significantly different from wt TRPC6. The properties of the double mutant M132T/N143S resulted in a similar current characteristic than the one observed for M132T (**Figure 2G–H**). M132T/N143S current rapidly reached a peak upon CCh application, and very little time-dependent inactivation was observed (**Figure 2H**). The averaged inward and outward current amplitudes, and the ratio of inward/outward current of wt TRPC6, N143S, M132T, and M132T/N143S are shown in **Figure 3** (n = 10 for each group).

**Figure 4:**
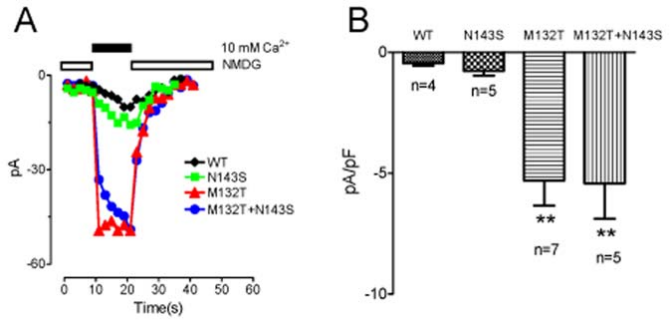
Increase in Ca^2+^ current in mutantsN143S, M132T and M132T/N143S. **A**: Inward current of wt TRPC6 and the mutants measured at −120 mV in the presence of 100 µM CCH. Inward current level was close to 0 pA level in NMDG solution, and increased after the cell was exposed to 10 mM calcium/NMDG solutions. Note the significant larger calcium current amplitude of M132T and M132T/N143S in comparison with wt TRPC6 and M143S. **B**: Averaged current amplitude of wt TRPC6, N143S, M132T, and M132T/N143S at −120 mV (mean±SEM, n = 4−7). (**: p<0.01 in comparison with wt TRPC6).

As TRPC6 is a non-selective cation channel, we further compared inward Ca^2+^ current amplitude of the mutants and the wt TRPC6. These experiments were conducted under the conditions that Ca^2+^ was the only permeate cation in the external solution. As shown in **Figure 4A**, the inward Ca^2+^ currents of M132T and M132T/N143S were significantly larger than those of wt TRPC6 and N143S. The smaller Ca^2+^ current amplitudes of N143S and wt TRPC6 are consistent with their transient activation feature as shown in **Figure 2B and 2F**. The mean inward calcium current of M132T and M132T/N143S was ∼10 fold larger than that of wt TRPC6 and N143S (**Figure 4B**).

## Discussion

This study represents one of the largest cohorts of FSGS families compatible with autosomal- dominant inheritance that was examined for *TRPC6* mutations. In one of 21 FSGS families that were compatible with autosomal-dominant segregation and screened for mutations, we identified a novel heterozygous disease-causing mutation in *TRPC6*, leading to early-onset FSGS in one of two affected individuals. The mutated ion channel causes a very significant increase in inward and outward currents and does not show channel inactivation. Only seven different *TRPC6* mutations in seven families have been identified so far, all leading to late-onset FSGS [9], [10], [12], whereas M132T mutation results in childhood FSGS.

We were able to show by whole cell current recordings, that M132T inward currents compared to the wild-type, were almost 7-fold increased after stimulation by Carbachol. The average outward and inward current amplitudes were 3 to 5-fold increased compared to wild-type, and inward Ca^2+^ current amplitude of the M132T mutant was almost 10-fold larger than wild type. We also observed that the M132T mutant did not show channel inactivation, in contrast to the wild-type channel whose current declined to 50% of the peak current level after reaching a peak level. Four of the previously reported *TRPC6* mutations (P112Q, R895C, E897K and Q899K) also showed gain of function in channel peak activity, leading to an increased Ca^2+^ influx and increased current amplitude upon activation [9], [10], [12]. The S270T and K874Stop mutants displayed increased time to channel inactivation and thus led to a gain of function in the TRPC6 cation channel. (10) In this study, we directly compared the different times to inactivation and average inward and outward channel currents of all published mutations, see **Table 1**. Interestingly, M132T mutant did not show any inactivation within 100 s, the S270T and K874X mutants showed slight inactivation of 10–15% within 79–90 s. In summary, these results show that all of the *TRPC6* mutations identified thus far lead to increased activity of TRPC6 ion channels, either by increasing current amplitude or by delaying channel inactivation.

Both the impaired inactivation of M132T, as well as the 10-fold increase in mean inward calcium current of M132T, provide an explanation for the early onset of SRNS in F505 IV-1. Our data supports the concept that mutations leading to a more pronounced increase in calcium influx, in this case by both direct increase in calcium influx and an impaired channel inactivation, also cause a more severe or possibly an earlier disease onset. As previous reported patients with disease-causing *TRPC6* mutations all manifested between the ages of 18 and 57, **see** **Table 1**, the consideration of mutated *TRPC6* was rejected for children with renal disease. Since this is the first time that a *TRPC6* mutation is shown to cause early-onset FSGS, it is required to consider mutated *TRPC6* as a possible cause for renal failure in children.

The N143S mutation, which was previously reported by Reiser et al, is located very close to M132T, within the same ankyrin repeat.(10) As of their close location, we expected channel kinetics of both mutants to be similar. Originally, N143S currents were described as not noticeably different from the wild-type TRPC6 peak channel activity.(10) In this study, the outward peak current of the N143S mutant again was not significantly different from the WT current, but caused a significant increase in the inward peak current amplitude (**Figure 3B**).

As M132T has such a strong Calcium current, and as the N143S and M132T mutations are located within the same ankyrin domain, we intended to investigate if two mutations in the N terminus would have a possible additive effect with respect to channel function. We therefore tested the effect of a double M132T/N143S mutant, hypothesizing an exponential increase of Calcium influx and time to channel inactivation. Current amplitudes of the double mutant M132T/N143S were however similar to the M132T mutant, but did not show a larger increase in current amplitude than the M132T mutant alone (**Figure 3**). Two mutations in the same domain cannot cause an exponential additive effect or change in channel kinetics and increase Calcium influx any further.

M132T leads to a comparably higher TRPC6 Calcium current than all other known mutations, even so one needs to carefully interpret this data considering that different TRPC6 channel stimulants have been used between the individual published studies. The high Calcium current of M132T in podocytes might serve as an explanation for the cause of an early onset FSGS disease in these patients.

The concept of calcium dose effect determining the time of disease onset is also supported by animal experiments where it was shown that transient overexpression of wild-type TRPC6 in murine glomeruli leads to rapid onset proteinuria and that elevated expression levels of TRPC6 are detected in different acquired human glomerular diseases [16]. While the idea of the amount of channeled calcium into podocytes as time-determining factor is certainly attractive, other contributing elements to TRPC6 disease onset need to be considered. For example, it was also shown that the P112Q TRPC6 mutant was more highly expressed at the cell surface and it was speculated that the altered subcellular trafficking could explain the enhanced channel activity [9], [17].

Evidence for TRPC6 playing an essential role in slit diaphragm signaling pathways has become robust over time. Interaction with podocin and nephrin in the slit diaphragm was shown [10], and binding to -actinin-1, -actinin-4 and calcineurin has also been reported [10], [18]. It is possible that TRPC6-mediated calcium influx regulates the actin cytoskeleton through pathways involving calcineurin. It was recently shown that the calcineurin-synaptopodin pathway could be a potential mediator of proteinuria. With calcineurin being a calcium-sensitive serin-threonine phosphatase, TRPC6 mediated calcium changes are an excellent candidate to directly affect cytoskeletal integrity of podocytes and thus glomerular barrier function [19]. The increased calcium influx through mutated TRPC6 into the cells might disrupt cytoskeletal response to physiological changes, thereby causing proteinuria [20]. Another pathophysiological explanation for proteinuria in patients with *TRPC6* mutations would be the dysfunction of mechanosensation in the slit diaphragm. Multiple TRP channel family members have been reported to be involved in mechanosensation, and altered TRPC6 channel function could influence podocyte sense for flow, stretch or pressure, thereby in turn affecting the reorganization of the podocyte actin cytoskeleton in response to physiological changes [20], [21]. The recent finding that podocin through cholesterol recruitment provides mechanosensitive properties to ion channels such as TRPC6, adds to the notion that the podocyte slit diaphragm relates information into podocyte foot processes [10], [22], [23]. Impaired cytoskeletal reorganization could be envisioned as a result of defective mechanosensation (e.g. through hyperactive TRPC6), which in turn leads to podocyte damage, podocyte loss and, ultimately, FSGS. In case of M132T mutation, this process would be enhanced.

Whatever the precise possibilities are that lead to increased compartmentalized calcium in podocyte foot processes, M132T is certainly one of them that together with other mechanisms requires us to consider large increase in TRPC6 channel currents as causative factors for early-onset FSGS. It is therefore advisable to screen already children in which FSGS segregates in an autosomal dominant mode, for mutations in *TRPC6*.

## Materials and Methods

### Patient and Data Recruitment

Within a worldwide cohort of 550 families with child-hood or adult-hood onset of FSGS, we selected 21 families with biopsy proven FSGS in affected individuals whom inheritance was consistent with an autosomal-dominant pattern. In affected individuals, we performed mutation analysis by direct exon sequencing for all 13 exons of *TRPC6*. All patients were also examined for mutations in *NPHS2* and exons 8 and 9 of *WT1* (Wilms' tumor-1) as these are a frequent cause of FSGS [24].

Human subjects research was approved by the University of Michigan Institutional Review Board and the Ethics Committee of the University of Freiburg, Germany. At least one affected individual per family had biopsy proven FSGS and nephrotic syndrome, diagnosed by nephrologists in specialized centers based on published criteria [25]. We obtained informed consent, detailed clinical and pedigree information by a standardized questionnaire completed available on www.renalgenes.org [26].

### Mutation Analysis

Genomic DNA was isolated from blood samples using the Puregene® DNA purification kit (Gentra, Minneapolis, MN) following the manufacturer's guidelines. Mutation analysis by direct exon sequencing was performed using exon-flanking primers. *TRPC6* exon primers are listed in **Table S2**. Exon primers for *NPHS2* and *WT1* have been published previously [26]. For sequence analysis the software SEQUENCHER^TM^ (Gene Codes, Ann Arbor, MI) was used. The published reference sequence of human *TRPC6* (NM_004621) was used as the relevant wild-type gene sequence. Sequencing of both strands was performed for the detected mutation. For the novel mutations their absence was demonstrated in >140 healthy individuals of matched ethnic origin by restriction-enzyme digest.

### Site-Directed Mutagenesis of the TRPC6 Plasmids

We introduced the M132T mutation into a full length human *TRPC6* cDNA clone by site-directed mutagenesis using the QuikChange Lightning Site Directed Mutagenesis Kit™ (Stratagene) according to the manufacturer's instructions. Mutagenic oligonucleotide primers containing the desired mutations were designed for amplification of the *TRPC6* cDNA template. cDNAs coding for non-tagged human wildtype TRPC6, FLAG-tagged human wildtype TRPC6, FLAG-tagged human TRPC6 carrying the N143S mutation [10], and a cDNA coding for the FLAG-tagged, dominant-negative human TRPC6 pore mutant all subcloned in the pcDNA3 vector (Invitrogen), served as templates for site-directed mutagenesis [27]. To confirm the identity of the N143/N143S and M132/M132T sites, both template and M132T-mutated plasmids were sequenced in quadruplicate.

### Electrophysiological Analysis of Mutant and Wild-Type TRPC6

HM1 cells (human embryonic kidney cells stably transfected with the M1 muscarinic receptor) were transiently transfected with wild-type (WT) TRPC6 or its mutants [28]. Electrophysiological recordings were conducted between 36–48 h after transfection. All patch-clamp experiments were performed at room temperature (20–25°C). Whole-cell currents were recorded using an Axopatch 200B amplifier. Patch electrodes were pulled from borosilicate glass and fire-polished to a resistance of ∼2 MΩ when filled with internal pipette solutions. Series resistance (R_s_) was compensated up to 90% to reduce series resistance errors to <5 mV. Cells in which R_s_ was >10 MΩ were discarded [29]. For whole cell currents recordings, voltage stimuli lasting 250 ms were delivered at 1- to 5-s intervals, with voltage ramps ranging from −120 to +100 mV. The holding potential was 0 mV. A fast perfusion system was used to exchange extracellular solutions, with complete solution exchange achieved in ∼1 to 3 s [30]. The internal pipette solution for whole cell current recordings contains (in mM) 145 Cs-methanesulfonate, 10 NaCl, 2 Mg-ATP, 0.2 Na-GTP, 1 EGTA, 0.38 CaCl_2_ and 10 HEPES buffer (pH 7.2 adjusted with CsOH). Free calcium concentration in the pipette solution was 100 nM as calculated by MaxChelator. The standard extracellular Tyrode's solution for whole cell current recording contains (mM): 145 NaCl, 5 KCl, 2 CaCl_2_, 1 MgCl_2_, 10 HEPES and 10 glucose; pH was adjusted to 7.4 with NaOH, and osmolarity was adjusted to ∼300 mOsm. N-methyl D-glucamine (NMDG) solution was prepared using (mM) 145 NMDG-Cl, 10 HEPES, and 10 glucose (pH 7.4). For some experiments, 10 mM Ca^2+^ was prepared in NMDG solution. The concentration of NMDG was reduced accordingly in order to keep the identical osmolarity.

### Statistical Analysis

Pooled data are presented as mean±SEM. Statistical comparisons were conducted by using one-way ANALYSIS of variance (ANOVA) and two-tailed t-test with Bonferroni correction; P<0.05 was chosen for statistical significance.

## Supporting Information

Table S1 Clinical characteristics of 21 families with FSGS compatible with autosomal dominant segregation-sensitive nephrotic syndrome.
(0.11 MB DOC)

Table S2 Primer sequences for all 13 exons of *TRPC6*.
(0.03 MB DOC)
