# Supplementary material for: A Novel TRPC6 Mutation That Causes Childhood FSGS
Source: PLoS One. 2009 Nov 10;4(11):e7771. doi: 10.1371/journal.pone.0007771 (PMC2777406; doi:10.1371/journal.pone.0007771)
Supplement: Table S1 — Clinical characteristics of 21 families with FSGS compatible with autosomal dominant segregation-sensitive nephrotic syndrome. (0.11 MB DOC) [file pone.0007771.s001.doc]

**Supplementary Table 1. Clinical characteristics of 21 families with FSGS compatible with autosomal dominant segregation**

| **Family #** | **Individual #^a^** | **Origin** | **Biopsy** | **Age at disease onset (years)** | **Phenotype** |
| --- | --- | --- | --- | --- | --- |
| **F721** | I-1 | Europe | - | 27 | SRNS |
|  | II-1 | Europe | FSGS | 12 | SRNS |
| **F940** | I-2 | - | FSGS | - | - |
|  | I-3 | - | FSGS | - | - |
|  | II-1 | - | - | 5 | - |
| **F505** | III-2 | Turkey | FSGS | 30 |  |
|  | IV-1 | Turkey | FSGS | 9 | SRNS |
| **F1019** | I-2 | - | FSGS | - | SRNS |
|  | II-1 | - | FSGS | 9 | SRNS |
|  | II-2 | - | FSGS | 11 | SRNS |
| **F1379** | I-2 | Turkey | FSGS | 22 | ESRD |
|  | II-1 | Turkey | - | 2 | - |
| **F1413** | I-1 | Europe | FSGS | ± 18 | - |
|  | II-1 | Europe | - | 17 | - |
| **F1421** | I-1 | Europe | Unspecific | 37 | - |
|  | II-1 | Europe | FSGS | 24 | - |
| **A263** | I-2 | Central Europe | - | 28 | - |
|  | II-1 | Central Europe | FSGS | 4 | - |
| **A572** | I-2 | Europe | - | - | - |
|  | II-1 | Europe | FSGS | 40 | - |
|  | II-2 | Europe | - | - | - |
| **A743** | I-1 | Turkey | - | - | ESRD |
|  | II-1 | Turkey | FSGS | 15 | - |
| **A828** | II-1 | Central Europe | - | ± 45 | ESRD, exitus |
|  | II-3 | Central Europe | - | ± 34 | ESRD, exitus |
|  | III-2 | Central Europe | FSGS | 19 | SRNS |
|  | III-3 | Central Europe | - | ± 33 | ESRD |
|  | IV-1 | Central Europe | FSGS | 14 | - |
| **A1467** | I-1 | Europe | FSGS | 18 |  |
|  | II-1 | Europe | FSGS | 17 | SRNS, ESRD |
| **A1498** | I-1 | USA | ? | 70 | SSNS |
|  | II-1 | USA |  | 10 | SSNS |
|  | IV-3 | USA | FSGS+mesangial proliferation | 5 | SRNS |
| **A1544** | I-2 | Europe | FSGS | 2 | - |
|  | II-1 | Europe | FSGS | 6 | - |
| **A1732** | I-2 | Europe | FSGS | 21 | - |
|  | II-1 | Europe | FSGS | 39 | - |
| **A1753** | II-2 | Caucasian | FSGS | 55 | - |
|  | III-1 | Caucasian | - | - | - |
| **A1757** | I-1 | Hispanic | ? | - | - |
|  | II-1 | Hispanic | FSGS | 13 | - |
|  | II-2 | Hispanic | FSGS | - | - |
| **A1849** | I-1 | Turkish |  | - | - |
|  | II-1 | Turkish | FSGS | 6 | SRNS |
| **A1927** | I-1 | Caucasian | - | 34 | SRNS |
|  | II-1 | Caucasian | FSGS | 11 | SRNS |
| **A2058** | I-2 | Hispanic | - | - | ESRD, exitus |
|  | II-1 | Hispanic | FSGS | 16 | SRNS, ESRD |
|  | II-3 | Hispanic | - | - |  |
|  | III-1 | Hispanic | - | 1 |  |
| **F691** | II-1 | Europe | - | - | - |
|  | III-1 | Europe | MCNS | - | SRNS |
