# Supplementary material for: A Novel TRPC6 Mutation That Causes Childhood FSGS
Source: PLoS One. 2009 Nov 10;4(11):e7771. doi: 10.1371/journal.pone.0007771 (PMC2777406; doi:10.1371/journal.pone.0007771)
Supplement: Table S2 — Primer sequences for all 13 exons of TRPC6. (0.03 MB DOC) [file pone.0007771.s002.doc]

**Supplementary Table 2. Primer sequences for all 13 exons of *TRPC6***

| **Exon #** | **5’ primer sequence** | **3’ primer sequence** |
| --- | --- | --- |
| **1** | CTGGAACTGCCCACTCGG | GTACACACGCGGGTTCAG |
| **2-1** | TCTTAGACGTGAAGTTCTTGGAATC | AACCGTGTCCCATCTTCATC |
| **2-2** | GGGGATGCTTTGCTTCTAGC | gaagctggtaaatACACCTTGACTC |
| **3** | ccttatttagcaccaacaagaacc | caagtctctgaagcatagtaaaacg |
| **4** | GAGATAAGATTTTTCCCCACTTAAAAC | TTTCATTGGAAAGCATGGAAC |
| **5** | CTCAGGAGATCATTGGAATGTG | CAGTGTCATTCAGTCCAACTGC |
| **6** | GAAGGGTTTTGTAATGTCAGGC | TGAGAATTGTGCAGTAACCG |
| **7** | CGAAAACCGGATTCTAACATAAC | CCAAAACATTATCCCATGGAC |
| **8** | TTTATACTCACTAATTTGCAGACACTAAAC | AGCAGTCCATGCTTTCATCC |
| **9** | CACAATGTTAAAAGCATCCCAG | AAAGGGATGTGGCATAGTGG |
| **10** | TGTTGGAGAATTAGACGAAATGG | TGCTTCTGAACATCTGTCCC |
| **11** | TGGCAGCCACAAAGTCTAAG | tgtaaggtttgccttttcaatg |
| **12** | TCCAAAGGGCTCACTACAGG | TGTACGCATCTCTGCAGCTC |
| **13** | TTTCCTCCTGTCCCACAGTC | TTAAAAGGTGGGCCCATTG |
